# Cardiac Magnetic Resonance for evaluating early outcomes of stem cell therapy in non-ischemic dilated cardiomyopathy

**DOI:** 10.1186/1532-429X-13-S1-P288

**Published:** 2011-02-02

**Authors:** Gurpreet S Gulati, Chesnal Arepalli, Sandeep Seth, Rajiv Narang, Balram Bhargava, Sujata Mohanty, Priya Jagia, Sanjiv Sharma, RK Ahuja, Balram Airan

**Affiliations:** 1 All India Institute of Medical Sciences, New Delhi, India; 2 All India Institute of Medical Sciences, Ansari Nagar, New Delhi, India, New Delhi, India

## Introduction

Non-ischemic dilated cardiomyopathy (DCM) patients on medical therapy have a high morbidity and mortality. The use of stem cell therapy in this patient subset has not been previously reported. Cardiac magnetic resonance (CMR) is the gold standard modality for quantifying ventricular function. We sought to evaluate the utility of CMR in assessing outcomes of stem cell therapy in DCM.

## Purpose

To report our experience with the use of CMR in assessing results of intracoronary stem cell therapy in non-ischemic DCM patients.

## Methods

Thirty seven patients with non-ischemic DCM underwent intracoronary injection of autologous bone marrow derived mononuclear cells. All patients gave informed consent for stem cell therapy and CMR studies. Mononuclear cells separated from aspirated bone marrow were injected into right and left coronary arteries. All patients had baseline CMR, while 10 underwent follow-up CMR (1.5 T, Magnetom Avanto, Siemens, Germany). Short axis cine-CMR was performed from the base to the apex of left ventricle (slice thickness 6-8 mm, inter-slice gap 2 mm). Images were analyzed for change in left ventricular (LV) and right ventricular (RV) ejection fraction (EF), corresponding ventricular volumes [end-diastolic (EDV), end-systolic (ESV) and stroke volume (SV)], and LV mass. Wilcoxon signed rank test was used to compare pre- and post-CMR results. Mann-Whitney U test was used to correlate change in functional class and CMR results

## Results

Of the 10 patients [eight males; mean age 48.6 years, range 30-64 yrs)], seven improved by at least 1 functional class, while three showed no change. Follow-up CMR was performed at 8.5±7.8 months. The LVEF (18.4±13% to 26.3±19%; p=.007), LVSV (29.9±13.4 ml to 52.8±30 ml; p=.013), RVEF (17±15.3% to 23.4±16.7%; p=.037) and RVSV (14.4±11.6 ml to 22.3±13 ml; p=.037) improved significantly. No significant change was noted for EDV, ESV or LV mass. Compared to those without functional improvement, patients with improvement showed greater favorable change in LVEF, LVSV, RVEF and RVSV. This difference was however, not statistically significant.

## Conclusions

CMR is accurate for assessing stem cell therapy outcomes in non-ischemic DCM. Our early results suggest that this treatment may be beneficial.

